# Behavior Matters—Cognitive Predictors of Survival in Amyotrophic Lateral Sclerosis

**DOI:** 10.1371/journal.pone.0057584

**Published:** 2013-02-27

**Authors:** William T. Hu, Matthew Shelnutt, Ashley Wilson, Nicole Yarab, Crystal Kelly, Murray Grossman, David J. Libon, Jaffar Khan, James J. Lah, Allan I. Levey, Jonathan Glass

**Affiliations:** 1 Department of Neurology, Emory University School of Medicine, Atlanta, Georgia, United States of America; 2 Center for Neurodegenerative Diseases, Emory University School of Medicine, Atlanta, Georgia, United States of America; 3 Alzheimer’s Disease Research Center, Emory University School of Medicine, Atlanta, Georgia, United States of America; 4 Department of Neurology, University of Pennsylvania, Philadelphia, Pennsylvania, United States of America; 5 Department of Neurology, Drexel University, Philadelphia, Pennsylvania, United States of America; Baylor College of Medicine, Jiao Tong University School of Medicine, United States of America

## Abstract

**Background:**

It is difficult to longitudinally characterize cognitive impairment in amyotrophic lateral sclerosis (ALS) due to motor deficits, and existing instruments aren’t comparable with assessments in other dementias.

**Methods:**

The ALS Brief Cognitive Assessment (ALS-BCA) was validated in 70 subjects (37 with ALS) who also underwent detailed neuropsychological analysis. Cognitive predictors for poor survival were then analyzed in a longitudinal cohort of 171 ALS patients.

**Results:**

The ALS-BCA was highly sensitive (90%) and specific (85%) for ALS-dementia (ALS-D). ALS-D patients had shorter overall survival, primarily due to the poor survival among ALS-D patients with disinhibited or apathetic behaviors after adjusting for demographic variables, ALS site of onset, medications, and supportive measures. ALS-D without behavioral changes was not a predictor of poor survival.

**Conclusion:**

ALS-D can present with or without prominent behavioral changes. Cognitive screening in ALS patients should focus on behavioral changes for prognosis, while non-behavioral cognitive impairments may impact quality of life without impacting survival.

## Introduction

Amyotrohpic lateral scleorsis (ALS) is a progressive neurodegenerative disorder that affects motor and extra-motor systems, and shares the pathologic hallmark of neuronal inclusions immunoreactive to TAR-DNA binding protein of ∼43 kD (TDP-43) with many cases of frontotemporal degeneration (FTD). [1], [2] About 15% of ALS patients may develop dementia (ALS-D) that resembles FTD, [3], [4] and FTD in ALS is associated with poor survival.[4]–[6] While detailed neuropsychological analysis can identify cognitive impairment in ALS in the research setting, such evaluation on a routine clinical basis is challenging because of the length of evaluation, patient fatigue, and motor-dependent tasks during testing. An abbreviated protocol that rapidly identifies ALS-D patients, tracks the severity of cognitive impairment over time, and has comparable performance in other dementias would significantly advance the understanding of ALS-D.

ALS patients can also develop mild cognitive impairment (ALS-CI) detectable on detailed neuropsychological analysis. Studies on ALS-CI and survival have generated conflicting findings, [4], [7] and the clinical utility of identifying ALS-CI remains unknown due to poor diagnostic sensitivity by available instruments. [8] We previously developed the Philadelphia Brief Assessment of Cognition that accurately detects and tracks cognitive deficits in FTD, [9] and PBAC is also used in the clinical diagnosis of mild cognitive impairment (MCI) and Alzheimer’s disease (AD). We have modified the PBAC into a 5-item ALS Brief Cognitive Assessment (ALS-BCA) by combining tests sensitive for executive and memory dysfunctions with a validated behavioral screen (Frontal Behavioral Inventory, FBI). [10] The ALS-BCA can be administered to ALS patients with variable dysarthria and limb weakness, and here we validated this instrument in 70 subjects (with and without ALS) who also underwent detailed neuropsychological analysis and diagnostic formulation through a consensus mechanism. We then applied the validated ALS-BCA to a large cohort of consecutive ALS patients to assess their executive function, language, memory, and behavior, and determined the differential effect of ALS-D, ALS-CI, and cognitive impairment subtype on survival in ALS.

## Methods

### 1. Subjects

Two cohorts of subjects were included in the current study, and all were diagnosed with possible, probable, or definite ALS. [11] This study was approved by the Emory University Institutional Review Board. The *validation* cohort included 37 ALS patients evaluated and longitudinally followed at the Emory ALS Center, and 43 healthy subjects longitudinally followed at the Emory Alzheimer’s Disease Research Center with normal cognition and behavior. All subjects independently underwent cognitive evaluation using the ALS-BCA and, on a separate day within 3 months, an in-depth neuro-cognitive evaluation consisting of structured neuropsychological analysis, language evaluation, and diagnostic formulation through a consensus mechanism. Written informed consents were obtained from all subjects or their legal representatives in the validation cohort. The *longitudinal* cohort consisted of 171 ALS patients serially followed at the Emory ALS Center on a regular interval (every 3–4 months), including 20 subjects from the validation cohort. There was no significant (p>.05) difference in age, disease duration, and cognitive performance between subjects from the validation cohort who were or were not part of the longitudinal analysis (data not shown). The ALS-BCA was administered as part of their clinical evaluation, and their ALS-BCA performance was retrospectively reviewed (WH, MS, and AW). As part of their initial clinical evaluation, all patients from the longitudinal cohort underwent structured interview for demographic information (including time and site of onset, family history of ALS/FTD), neurological examination, functional assessment using the revised ALS Functional Rating Scale (ALSFRS-R), [12] breathing assessment for forced expiratory volume (FEV) as a percentage of predictive value, electromyography to determine denervation, and blood tests and MRI to exclude other causes of progressive motor symptoms. All subsequent visits were reviewed (WH) to determine whether a patient was started on ALS therapies, including riluzole, non-invasive positive pressure ventilation (NPPV), and percutaneous endoscopic gastrostomy (PEG). Death information was obtained from clinical records for 59 patients and from local obituaries for 2 patients.

### 2. Cognitive Evaluation

**Figure 1:**
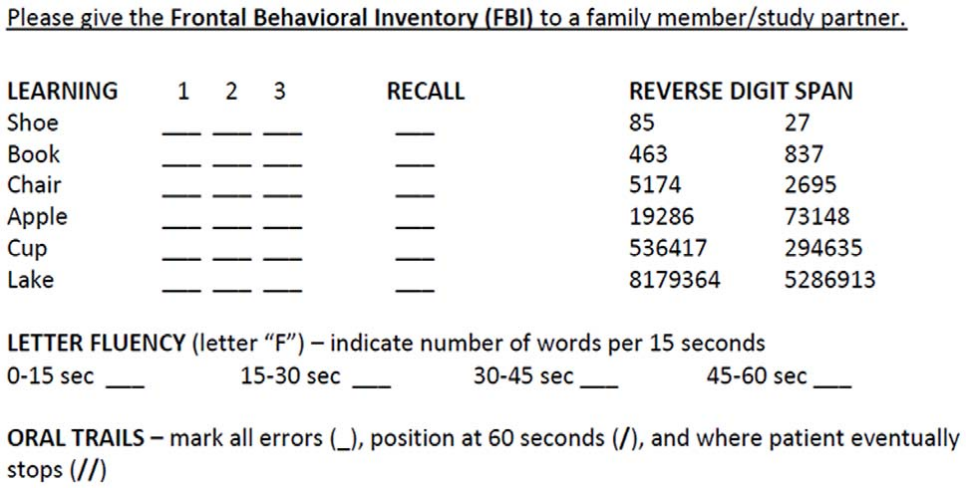
ALS Brief Cognitive Assessment (ALS-BCA). For patients who have mild to moderate dysarthria, they are instructed to proceed with word list learning, reverse digit span, word list recall, letter fluency, and oral trails (see Methods). For patients who have severe dysarthria or anarthria, a modified ALS-BCA is administered as follows: for learning and recall, subjects write or type the first letter of each word during learning, and write down the whole words during delayed free recall; for reverse digit span, subjects write down the numbers in reverse order; for letter-guided fluency, subjects write as many F-words as possible until they give up or until 16 words have been generated. Oral trails is omitted for these patients.

The ALS-BCA is a 5-item assessment that evaluates a subject’s executive functions (working memory, set-shifting), frontally-mediated language function, delayed verbal recall, and behaviors common in FTD (Figure 1). Four items were derived from the PBAC for their association with FTD: [9], [13] for executive function, working memory and the capacity for effective mental manipulation was assessed with the Reverse Digit Span subtest where subjects are given trials consisting of 2 to 7 numbers and the Oral Trail Making Test [14] where subjects are asked to verbally alternate between numbers and letters (i.e., 1-A-2-B, etc.); for frontally-mediated language function, subjects are asked to generate as many words as possible that begin with “F” within one minute; for delayed free recall, subjects are asked to repeat a list of six words for three separate trials, and then recall all words after a brief delay. For anarthric patients, a modified protocol omitting oral trails is performed (Figure 1). Performance of healthy subjects from the validation cohort was used to establish a norm for the ALS-BCA, with −1.5 standard deviation below the mean as the cut-off for abnormal scores. ALS subjects had abnormal performance if they had the following: delayed recall at 40% or less of Trial 3 learning, reverse digit span at 3 or below, letter-guided fluency of 8 words or less in one minute, and oral trails performance of 42 or less. Lastly, for all subjects, a behavioral questionnaire (FBI) [10] is administered to the caregiver to assess behavioral and personality changes from baseline (with permission from Dr. A. Kertesz). The FBI is a 24-item caregiver questionnaire that rates the severity of behavioral symptoms common in FTD, including 12 items addressing disinhibited behaviors and 12 items addressing negative behaviors. Each item is rated from 0 to 3 according to severity (0 = never, 1 = mild or occasional, 2 = moderate, 3 = severe or very frequent), and previous work has shown that FTD patients have greater total scores than patients with AD, depression, or vascular dementia. [15].

Subjects in the validation cohort underwent detailed neuropsychological analysis according to the procedures of the Alzheimer Disease Centers Uniform Data Set, [16] including tests of attention and executive functions (forward and reverse digit span, [17] Trail Making Test A & B, [18] similarities [19]), verbal encoding and retrieval, language (category fluency, [20] and the 30-item Boston Naming Test for confrontation naming [21]). Tests for letter-guided fluency, [22] visual encoding and retrieval (Brief Visuospatial Memory Test-Revised), [23] and visual spatial relations (judgment of line orientation test) [24] were also performed according to standard protocols. The raw score for each patient was converted to age-, gender-, and education-adjusted Z-scores, and a Z-score at or below −1.5 was considered abnormal. Findings from the clinical history, neurological examination (including degree of dysarthria and limb weakness), and neuropsychological analysis were then used to derive a consensus diagnosis of normal cognition, ALS-CI, or ALS-D. Specifically, neurologists and one neuropsychologist will assess the history, number of abnormal cognitive tests and domains for each subject, determine the relationship between the motor-dependent aspects of each test and individual patients’ motor deficits, functional deficits from motor weakness, and overall functional deficits to derive the diagnosis. ALS-CI was diagnosed when there were objective cognitive deficits but the deficits were not severe enough to cause functional deficits independent of the motor deficits, and ALS-D was diagnosed when there were sufficient cognitive deficits independent of motor findings to result in a functional decline from baseline. In the validation phase, we did not include patients who were completely mute or unable to write as they could not participate fully in traditional neuropsychological protocols.

### 3. Statistical Analysis

All statistical analysis using de-identified data was performed in SPSS 20.0 (Chicago, IL). Baseline comparisons between subjects with normal cognition, ALS-CI, and ALS-D were performed by Chi-squared tests for categorical variables and analysis of variance for continuous variables. Sensitivity and specificity of ALS-BCA for ALS-CI and ALS-D were determined by receiver-operating characteristics (ROC) curve analysis. Survival analysis was performed first by Kaplan-Meier analysis for the effect of ALS-CI or ALS-D, with p<0.01 as a threshold for significance at the ALS-BCA subtest level to adjust for multiple comparisons. Tests linked to poor survival were then analyzed in a Cox proportional hazards analysis to adjust for contribution from other factors, with age, gender, disease duration, and bulbar- vs. limb-onset were entered as fixed variables in the first step, and abnormal ALS-BCA test, FEV, rilutek use, NPPV use, PEG use, and family history entered in a forward likelihood ratio fashion.

## Results

### 1. Validation Cohort

**Figure 2:**
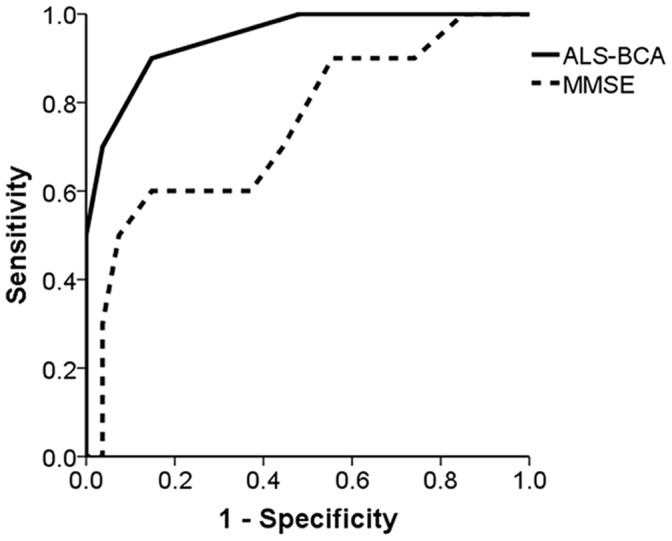
Receiver operating characteristics curve for ALS-BCA and MMSE for the diagnosis of ALS-D. Areas under the curve are 0.946 for ALS-BCA and 0.746 for MMSE.

In the validation cohort (n = 70, Table 1), ALS-D subjects were older and were more cognitive impaired than other ALS subjects. When all subjects were administered ALS-BCA, 2 or more abnormal subtest scores was associated with 90% sensitivity and 85.2% specificity for the prediction of ALS-D (area under the curve of 0.946, Figure 2), compared to 70% sensitivity and 55.6% specificity for a MMSE score below 26 (area under the curve of 0.746). The one ALS-D subject with fewer than 2 abnormal ALS-BCA subtests was a 40-year-old man with definite ALS, a FBI score of 38, and impaired confrontation naming (Z-score −1.60). He was given the consensus diagnosis behavioral variant FTD based on behavior changes without a psychiatric or medical explanation. For any clinically significant cognitive impairment (ALS-CI or ALS-D), 1 or more abnormal subtest scores on ALS-BCA had modest sensitivity (79.2%; 100% for ALS-D and 64% for ALS-CI) and specificity (69.2%) compared to MMSE score below 27 (sensitivity 62.5%, specificity 69.2%).

**Table 1:** Baseline characteristics of the validation cohort, including healthy control subjects and ALS patients according cognitive status of normal cognition, cognitive impairment but no dementia (ALS-CI), or dementia (ALS-D).

	Healthy Control (n = 44)	ALS-Normal Cognition(n = 13)	ALS-CI(n = 14)	ALS-D(n = 10)	p(between ALS groups)
Male (percentage)	18 (41%)	9 (69%)	11 (79%)	9 (90%)	0.487
Age, yr (SD)	68.3 (19.6)	54.3 (8.9)	60.9 (10.9)	65.0 (11.1)	0.059
Education, yr (SD)	16.9 (2.1)	15.0 (2.2)	15.4 (2.9)	16.3 (2.0)	0.454
**Consensus Diagnosis**					
Normal	44	13	0	0	
Executive impairment	0	0	8	8	
Language impairment	0	0	6	2	
**ALS-BCA scores**					
All normal	39 (89%)	9 (69%)	5 (36%)	0	
1 abnormal score	4 (9%)	3 (23%)	6 (43%)	1 (10%)	
2 abnormal scores	1 (2%)	1 (8%)	2 (14%)	3 (30%)	0.008
3 abnormal scores	0	0	1 (7%)	1 (10%)	
4 abnormal scores	0	0	0	4 (40%)	
5 abnormal scores	0	0	0	1 (10%)	
**Neuropsychological analysis**					
MMSE	29.0 (1.4)	25.3 (7.8)	26.1 (2.8)	22.6 (5.0)	0.305
Z, Logical memory, immediate	0.20 (0.81)	0.25 (0.80)	−1.15 (0.74)*	−2.55 (1.90)†	<0.001
Z, Logical memory, delayed	0.22 (0.91)	0.12 (0.56)	−1.12 (0.68)*	−2.27 (1.89)†	<0.001
Z, Word list delayed recall	0.42 (0.75)	0.76 (0.38)	−0.25 (0.96)	−2.27 (2.05)†	<0.001
Forward digit span	9.22 (5.37)	7.08 (2.84)	7.07 (2.16)	5.90 (3.03)	0.499
Reverse digit span	6.92 (2.07)	6.08 (1.38)	5.71 (2.46)	3.11 (1.62)*	0.003
Z, Letter-guided fluency	−0.02 (0.92)	0.24 (0.49)	−0.76 (0.87)*	−2.39 (1.69)†	<0.001
Z, Category fluency	−0.12 (1.03)	−0.08 (0.65)	−0.76 (.90)	−2.50 (1.77)*	<0.001
Z, Confrontation Naming	0.79 (1.08)	0.15 (0.96)	−0.42 (1.25)	−1.01 (1.17)*	0.063
Z, Digit symbol test	0.84 (0.81)	−0.27 (0.65)	0 (1.01)	−1.24 (1.03)	0.127
Z, TMT A	0.34 (1.16)	−0.70 (1.28)	−0.97 (0.96)	−2.23 (2.22)	0.172
Z, TMT B	0.01 (1.05)	0.07 (1.33)	−1.00 (0.96)	−2.13 (2.31)	0.072
Z, Judgment of line orientation	0.44 (0.87)	0.29 (0.90)	0.67 (1.16)	−0.34 (1.43)	0.475
Geriatric Dementia Score	1.10 (1.60)	4.27 (3.95)	3.85 (2.97)	4.60 (3.78)	0.908

ALS-BCA: ALS Brief Cognitive Assessment; MMSE: Mini-Mental Status Examination score; TMT: Trail making test;

* different from ALS subjects with normal cognition;

† different from ALS subjects with normal cognition and ALS-CI.

### 2. Longitudinal Cohort

The longitudinal cohort for survival analysis contained 171 consecutive ALS patients with a median follow-up of 9 months. At baseline, 25% of the ALS patients had impaired performance on two or more subtests (ALS-D), and 27% of the ALS patients had impaired performance on only one subtest (ALS-CI, Table 2). Impairments in letter-guided fluency (67% in ALS-D and 47% in ALS-CI) and reverse digit span (70% in ALS-D, 19% in ALS-CI) were common, with impairments on Oral Trails more common in ALS-D (70%) than ALS-CI (8%). Abnormal behavior was uncommon in both ALS-D (19%) and ALS-CI (11%). As a group, ALS-D patients were older at baseline, but otherwise were similar to the two other ALS groups according to gender, disease duration, site of onset, clinical ALS diagnosis, [11] family history, ALS-FRS, FEV, and use supportive measures (riluzole, NPPV, PEG).

**Table 2:** Baseline characteristics of the longitudinal cohort according to number of abnormal tests on ALS-BCA.

Abnormal ALS-BCA tests	None (n = 81 )	1 (n = 47)	2 or more (n = 43)	p
Male (%)	51 (63%)	26 (55%)	31 (72%)	0.257
Age (SD), years	59.1 (9.9)	58.8 (12.0)	65.4 (12.1)	0.005
Disease duration (SD), years	2.9 (2.5)	2.8 (2.4)	3.1 (2.6)	0.806
Family history of ALS/FTD(%)	5 (6%)	1 (2%)	2 (5%)	0.579
ALSFRS-R (SD)	32.2 (8.2)	30.9 (8.9)	30.3 (9.7)	0.505
% FEV predicted (SD)	76.0 (26.7)	68.9 (25.2)	67.0 (30.8)	0.152
Bubar-onset (%)	17 (21%)	11 (23%)	13 (30%)	0.474
Clinical ALS diagnosis				0.552
Definite ALS (%)	41 (50%)	23 (49%)	21 (49%)	
Probable ALS, EMG+ (%)	20 (25%)	14 (30%)	13 (30%)	
Probable ALS (%)	13 (16%)	8 (17%)	3 (7%)	
Possible ALS (%)	7 (9%)	2 (4%)	6 (14%)	
ALS-BCA performance				
Z, delayed recall (SD)	0.01 (0.69)	−0.39 (1.07)	−0.84 (1.18)	<0.001
Z, reverse digit span (SD)	0.14 (0.94)	−0.34 (1.02)	−1.49 (0.94)	<0.001
Z, letter-guided fluency (SD)	0.13 (1.15)	−0.96 (1.21)	−1.94 (1.14)	<0.001
Z, trails (SD)	0.22 (0.41)	−0.39 (1.91)	−4.70 (3.46)	<0.001
FBI	5.9 (5.9)	10.3 (11.9)	13.7 (11.2)	<0.001
Follow-up (SD), months	9.8 (4.6)	9.8 (5.8)	9.0 (5.4)	0.723
Riluzole use (%)	42 (52%)	21 (45%)	18 (42%)	0.519
NPPV use (%)	39 (48%)	25 (53%)	22 (51%)	0.852
PEG use (%)	12 (15%)	3 (6%)	9 (21%)	0.134

**Figure 3:**
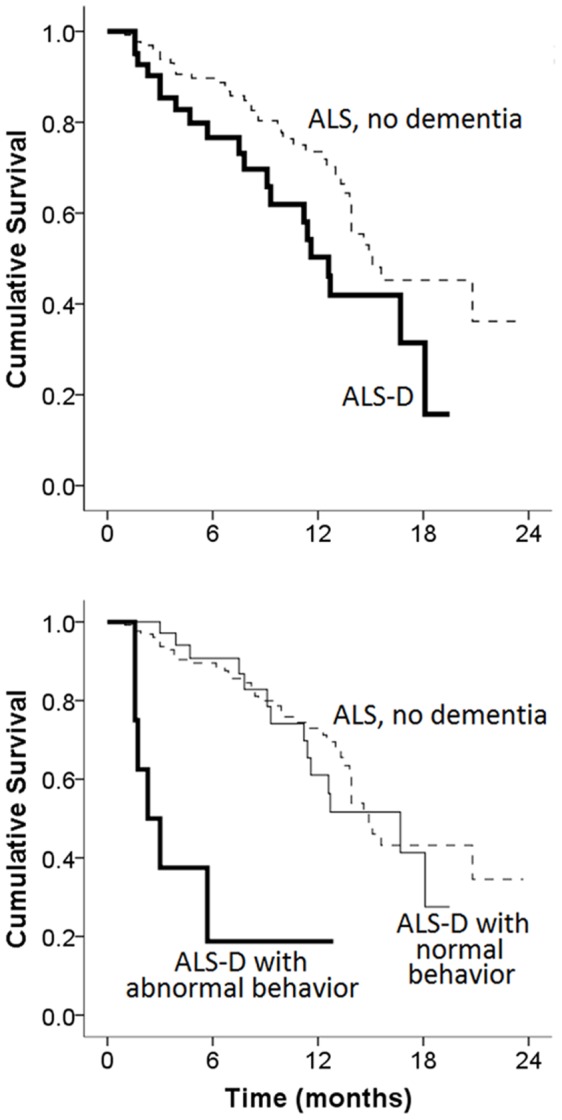
Kaplan-Meier analysis of the effects of dementia and abnormal behavior on ALS survival. Top: ALS-dementia (ALS-D) patients have poorer survival than non-demented ALS patients (p = 0.03). Bottom: ALS-D patients with abnormal behavior had significantly worse survival than ALS-D patients with normal behavior and non-demented ALS patients (p<0.001).

61 ALS patients (36%) died during a median follow-up of 9 months. ALS-D patients had worse overall survival than non-demented ALS patients (p = 0.03, Figure 3), but ALS-CI was not associated with poorer survival compared to ALS patients with normal cognition (p = 0.463). 15 patients underwent neuropathologic evaluation after death, including 6 with normal cognition, 2 with ALS-CI, and 7 with ALS-D. Among these, 9/15 had diffuse cortical FTLD-TDP pathology, and 8/15 had some form of neuritic plaque and neurofibrillary tangle pathology (1 with probable AD, 5 with possible AD, and 2 with isolated tau pathology). There was no significant difference in cortical FTLD-TDP pathology and AD-related pathology according to cognitive or behavioral abnormalities. Classification of ALS-CI patients into dysexecutive (abnormal performance in reverse digit span, letter-guided fluency, or oral trails), amnestic (abnormal retrieval of encoded verbal information), and behavioral (abnormal FBI) subtypes did not reveal any subtype to be associated with worse survival (p = 0.482).

To explore if one ALS-BCA subtest was the strongest predictor of poor survival, we performed Kaplan-Meier analysis on the longitudinal cohort according to normal or impaired performance in one of the five subtests (free verbal recall, reverse digit span, letter-guided fluency, oral trails, behavior). Abnormal behavior (FBI score of 23 or more) was strongly associated with poor survival (p<0.001), but survival was not influenced by impaired performance in delayed free recall (p = 0.130), reverse digit span (p = 0.08), letter-guided fluency (0.185), or oral trails (p = 0.123). When only ALS-D patients were analyzed, patients with abnormal FBI scores had significantly worse survival than patients with normal behaviors. After this dichotomization, ALS-D with normal behavior was no longer associated with poor survival (Figure 3). This was confirmed in a multi-variate Cox proportional hazards analysis (Statistical Analysis; Table 3), and entering additional variables such as the Clinical ALS diagnosis (definite, probable with laboratory support, probable, possible) resulted similar outcomes. ALS-D patients with abnormal behaviors (n = 8) scored higher on both the disinhibition and negative behavioral scores than ALS-D patients with normal behaviors (n = 35; mean score 10.1 vs. 2.69 for disinihibition, 21.5 vs. 6.89 for negative behavior, p<0.001 for each case). Further analysis revealed that compared to ALS-D patients with normal FBI scores, ALS-D patients with abnormal FBI scores additionally had shorter disease duration prior to cognitive assessment (1.94 yr vs. 3.38 yr, p = 0.02), but were otherwise similar in age, gender, site of onset, family history, ALS-FRS, riluzole use, NPPV use, and PEG use. Dividing the ALS-D patients with normal behaviors into dysexecutive, amnestic, and language subtypes did not reveal any impact of the main deficit type on survival.

**Table 3:** Cox proportional hazards model for abnormal scores on the Frontal Behavioral Inventory (FBI).

Factor	Exp (B) (95% confidence interval)	p
Age	1.031 (1.007–1.055)	0.011
Gender	0.552 (0.314–0.972)	0.039
Bulbar onset	0.588 (0.314–1.100)	0.097
Disease duration (years)	0.652 (0.538–0.791)	<0.001
ALSFRS-R	0.920 (0.886–0.954)	<0.001
Abnormal FBI	0.334 (0.145–0.770)	0.009

Age, gender, bulbar onset, and disease duration were entered as fixed variables; ALS-FRS, abnormal FBI, ALS-D, riluzole use, and PEG use were entered in a forward likelihood fashion.

## Discussion

A number of tools have been proposed for the diagnosis of ALS-D and ALS-CI given the overlap between ALS-D and FTD, but they serve primarily as screening instruments and are not in regular use in cognitive research. Here we modified a previously validated tool for FTD to derive the ALS-BCA, and demonstrated its high sensitivity and specificity for ALS-D in a large validation cohort. The applicability of ALS-BCA and the broader PBAC will enable the comparative analysis of cognitive decline between ALS and other motor-sparing disorders including FTD and AD. Furthermore, we prospectively used ALS-BCA in a larger longitudinal cohort to show ALS-D to be quite common, and the poor survival associated with ALS-D is primarily due to patients with abnormal behaviors while dementia without behavioral abnormalities did not significantly influence overall survival in ALS.

Cognitive impairment in ALS has recently become a focus of detailed clinical and neuropsychological research. [2], [25], [26] Consensus criteria have been proposed for frontotemporal dysfunctions in ALS, [27] but the significance of making the diagnosis of ALS-CI or ALS-D in a disorder with high morbidity and mortality is unknown. Complicating matters further in the syndromic characterization of ALS patients with cognitive impairment are the evolving definition of behavioral variant FTD [28]–[31] and feature overlaps between behavioral and motor deficits (e.g., inertia due to behavior vs. weakness). While detailed neuropsychological evaluation in ALS centers with local neuropsychological expertise is possible, [5], [7], [32] such practice is often not practical in most ALS clinics. Among traditional screening tools, the MMSE is poorly sensitive for ALS-D and ALS-CI, [33], [34] and the sensitivity of Montreal Cognitive Assessment [35] and Addenbrooke’s Cognitive Examination [36] for ALS-D and ALS-CI are unknown. Behaviorally, the Cambridge Behavioral Inventory, [37] the FBI, [38]and the Frontal Assessment Battery [33], [34] have all been used in ALS populations, but the small proportion of ALS-D patients in our study with highly abnormal behaviors suggests that behavioral-only screening is insensitive for ALS-D and ALS-CI. Similar to the ALS-CBS™, [8] ALS-BCA sought to determine behavioral and non-behavioral cognitive changes in ALS patients. Unlike ALS-CBS™, ALS-BCA has the unique property of using number of abnormal subtests instead of overall additive score as a threshold for ALS-CI or ALS-D, which resulted in improved sensitivity for ALS-CI (64%) compared to ALS-CBS™ (25%). [8] This approach is no different from the formulation of MCI-AD subtypes (single domain vs. multi-domain), and retains information on the number and type of abnormal subtests. Furthermore, the public domain ALS-BCA can be used to track longitudinal outcome according to absolute Z-scores or categorical diagnoses (progression, stability, reversion), and the increasing use of public domain PBAC in dementia clinics for MCI, AD, and FTD makes ALS-BCA the ideal clinical and research tool for comparative studies. [13].

The phenotypic information derived from the ALS-BCA also enabled us to conclude that ALS-D patients with abnormal behaviors, but not ALS-D patients with normal behaviors, have the poorest survival. This differs qualitatively from prior studies which mostly found FTD to be associated with poor survival in ALS. For example, one such study [5] showed that ALS patients with sufficient executive and behavioral changes for the diagnosis of behavioral variant FTD by Neary criteria [28] had poorer survival than other ALS patients, [5] and Neary criteria for behavioral variant FTD were used to detect demented ALS subjects in two subsequent studies. [4], [6] However, Neary criteria have limited sensitivity in early FTD [30] and its sensitivity in ALS-FTD or ALS-D is unknown. This limitation may have led to the identification of only ALS patients with prominent behavioral changes as ALS-D subjects in prior studies while underestimating the overall prevalence of ALS-D. In other words, ALS-FTD patients in previous studies are likely equivalent to ALS-D patients in our current study with abnormal behaviors, while ALS-D patients with normal behaviors were not considered FTD-like. Therefore, it may be difficult to directly compare the prevalence of ALS-D reported in the current survival study (25%) with other survival studies that employed the Neary criteria, but the reported prevalence here is similar to that reported in a study using detailed neuropsychological criteria. [32] Our finding that behavior is a more important predictor of survival than the diagnosis of ALS-D or ALS-CI is crucial, as behavioral changes can be reliably reported by caregivers independent of the patients’ abilities to undergo cognitive testing. At the same time, it is not immediately apparent why ALS-D with normal behavior is not associated with poor survival. One possibility is that ALS-D patients with abnormal behaviors have more severe neuropathology, and work is ongoing to determine whether the distribution and severity of TDP-43 pathology is associated with behavioral abnormalities. Another possible explanation may be impairment in other higher cortical functions not routinely tested even in detailed neuropscyhological analysis such as theory of mind. [39] Impairments in theory of mind are thought to underlie some of the behavioral abnormalities in FTD, [40]–[42] and abnormal behaviors in ALS-D may indicate yet more severe neurodegeneration or network abnormalities [43] associated with poorer survival. If this is the case, the neuronal neuropathology and measures of cortical function may not significantly differ between ALS-D patients with and without behavioral changes, but the glial neuropathology and large-scale brain network impairment may be more prominent in ALS-D patients with abnormal behaviors. Future studies in living patients using diffusion tensor imaging or functional MRI may be useful to detect network dysfunction in ALS-D.

The phenotypic and quantitative nature of ALS-BCA additionally allowed us to study the impact of ALS-CI on survival. In the longitudinal cohort, executive dysfunction accounted for approximately 75% of all ALS-CI. Similar to two prior studies, [32], [44] we did not find ALS-CI to be a predictor of poor survival, although the sensitivity of ALS-BCA for ALS-CI only (64%) is still under 80% despite the relative improvement from ALS-Cognitive Behavioral Screen (ALS-CBS)™ (25%). At the same time, ALS-BCA It also remains unclear if ALS-CI as a diagnosis or a particular ALS-CI syndrome may be sufficient to interfere with activities of daily living and use of adaptive equipments (PEG, NPPV). This raises the question of whether ALS-CI carries the same clinical significance as MCI due to AD. There were too few conversions (from ALS-CI to ALS-D) in the current study to determine whether ALS-CI is a prodromal phase to ALS-D, and ALS-CI may represent a heterogeneous group of disorders with cognitive impairment stemming from extra-motor ALS pathology, hypoxia, or nutritional factors. As we continue to gather longitudinal cognitive and clinical data on this and other cohorts, the etiology and outcome associated with ALS-CI as a diagnostic entity can be more directly addressed.

While the ALS-BCA has high diagnostic accuracy for ALS-D, this study has a number of limitations. The ALS-BCA is geared towards common deficits in FTD and AD, but does not contain subtests that evaluate parietal functions. While visual-spatial dysfunction is more common in FTD cases associated with tau pathology, [45] confrontation naming is often impaired in semantic variant of primary progressive aphasia which shares TDP-43 pathology with ALS (albeit with different TDP-43 subtypes). [46] ALS-BCA in the current study was administered as patients were initially evaluated at the ALS Clinic, but it was difficult to determine retrospectively whether abnormal cognition had been present since disease onset even though we adjusted for disease duration in our multi-variate model. The current cohort may not be sufficiently powered to detect the effects of ALS-D with normal behavior and ALS-CI on survival, and the ALS-D with abnormal behavior cohort was limited in size to determine the effect of specific behaviors (e.g., ritualistic behavior vs. apathy) on survival. Lastly, only a proportion of ALS subjects had neuropathologic evaluation, and there were too few cases for detailed examination on the pathologic differences between ALS-CI, ALS-D with normal behavior, and ALS-D with abnormal behaviors. Prospective studies examining the severity and distribution of TDP-43 pathology are on-going to address the possible connection between pathologic burden, executive functions, and behavior. Nevertheless, ALS-BCA can rapidly and accurately identify cognitive dysfunction in a busy multi-disciplinary ALS clinic, and can be used to longitudinally study the significance of isolated cognitive impairment on ALS patients and caregivers.
